# Plane Wave SH_0_ Piezoceramic Transduction Optimized Using Geometrical Parameters

**DOI:** 10.3390/s18020542

**Published:** 2018-02-10

**Authors:** Guillaume Boivin, Martin Viens, Pierre Belanger

**Affiliations:** Département de génie mécanique, École de technologie supérieure, 1100 rue Notre-Dame Ouest, Montréal, QC H3C 1K3, Canada; martin.viens@etsmtl.ca (M.V.); pierre.belanger@etsmtl.ca (P.B.)

**Keywords:** ultrasound, structural health monitoring, guided waves, shear horizontal waves, transducer, piezoceramic

## Abstract

Structural health monitoring is a prominent alternative to the scheduled maintenance of safety-critical components. The nondispersive nature as well as the through-thickness mode shape of the fundamental shear horizontal guided wave mode (SH${}_{0}$) make it a particularly attractive candidate for ultrasonic guided wave structural health monitoring. However, plane wave excitation of SH${}_{0}$ at a high level of purity remains challenging because of the existence of the fundamental Lamb modes (A${}_{0}$ and S${}_{0}$) below the cutoff frequency thickness product of high-order modes. This paper presents a piezoelectric transducer concept optimized for plane SH${}_{0}$ wave transduction based on the transducer geometry. The transducer parameter exploration was initially performed using a simple analytical model. A 3D multiphysics finite element model was then used to refine the transducer design. Finally, an experimental validation was conducted with a 3D laser Doppler vibrometer system. The analytical model, the finite element model, and the experimental measurement showed excellent agreement. The modal selectivity of SH${}_{0}$ within a 20${}^{\circ }$ beam opening angle at the design frequency of 425 kHz in a 1.59 mm aluminum plate was 23 dB, and the angle of the 6 dB wavefront was 86${}^{\circ }$.

## 1. Introduction

Structural health monitoring (SHM) is transforming multiple industries in shifting from scheduled maintenance to condition-based maintenance. In SHM, transducers are permanently attached to the structure of interest in order to periodically inspect it. In the aerospace industry, a viable SHM system will need to maximize the coverage from a minimum number of transducer locations. Moreover, each transducer must have the minimum footprint and weight. The ability of ultrasonic guided waves to propagate over distances on the order of a few meters with minimal attenuation makes them an attractive option in the development of an active SHM system [1,2,3]. Unique properties of the fundamental shear horizontal mode (SH${}_{0}$) have recently attracted attention from the scientific community in nondestructive testing (NDT) and SHM [4,5,6]. SH${}_{0}$ is the only nondispersive ultrasonic guided wave mode, and it does not have attenuation due to fluid loading [7]. The nondispersive nature of SH${}_{0}$ combined with a broadband transducer design may improve the detection resolution because short input signals can be used. Moreover, SH${}_{0}$ will not convert to other guided wave modes when interacting with defects or features perpendicular to the direction of propagation, therefore leading to increased sensitivity [8].

The generation of plane SH${}_{0}$ wave is of particular interest in the NDT inspection of welded, riveted, or bonded joints [5,9,10]. In order to adapt those NDT methods for the continuous monitoring of joints, a compact, lightweight, rugged transducer is required. The scientific community has so far demonstrated concepts using polyvinylidene difluoride (PVDF) interdigital transducers [11], electromagnetic acoustic transducers (EMATs) [12,13], and piezoceramic transducers [4,14,15]. However, the transduction efficiency of PVDF interdigital transducers is poor, and EMATs are typically bulky and therefore not well adapted to permanent installations. Moreover, EMATs will not work on composite structures unless a magnetostrictive patch is first bonded to the structure [16]. Although piezoceramic transducers were used in the past for the generation and detection of SH${}_{0}$, no particular effort was put towards optimizing their modal selectivity [4,15,17,18,19]. Previous papers failed to combine crystal orientation, transducer geometry, and appropriate vibrational modes together to generate pure SH${}_{0}$ without other modes.

This paper therefore focuses on the development of a lightweight plane wave SH${}_{0}$ piezoceramic transducer with high modal selectivity using geometrical considerations. The first section details the piezoelectricity theoretical background—including vibrational mechanisms—required to select the most appropriate material for the generation of the SH${}_{0}$ mode. The two geometric parameters used to maximize the selectivity of the SH${}_{0}$ mode are also discussed in the first section. The second section presents the analytical wave propagation model used to explore the geometrical parameters, which were then refined using finite element simulations. Finally, the third section presents the experimental validation of the proposed design.

## 2. Theoretical Background

**Figure 1:**
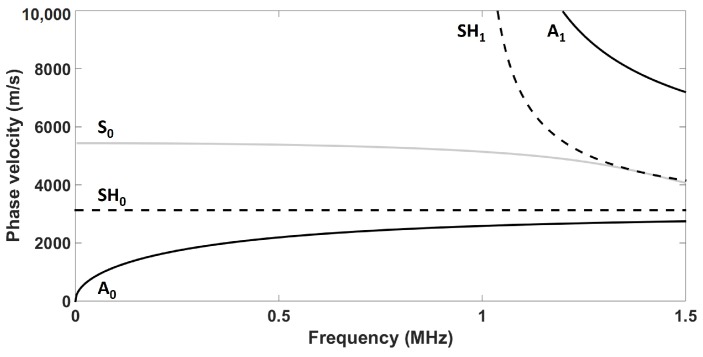
Phase velocity dispersion curves in a 1.59 mm aluminum plate (E = 70.8 GPa, ν = 0.34, and ρ = 2700 kg/m${}^{3}$). The solid black lines correspond to the $A_{0}$ and $A_{1}$ modes, the solid light-gray line corresponds to the $S_{0}$ mode, and the dashed black lines correspond to the ${\mathrm{SH}}_{0}$ and ${\mathrm{SH}}_{1}$ modes.

Ultrasonic guided waves are mechanical stress waves that can propagate in solid plate-like media. These waves can be grouped into two classes—namely, Lamb waves (symmetrical and antisymmetrical modes (A and S)) and SH waves. For each class, fundamental and high-order modes can exist. Fundamental modes exist at any frequency thickness product, while high-order modes appear at a specific frequency thickness product, known as the cutoff frequency thickness product [20] (p. 108). Unlike bulk ultrasonic waves in isotropic solids, the velocity of ultrasonic guided waves varies with the frequency because of dispersion. The study presented in this paper was conducted on a 1.59-mm aluminum plate, as thin aluminum structures are particularly relevant to aerospace SHM. However, the results presented in this paper could easily be adapted to other plate thicknesses or materials by using the design rules described below. Figure 1 presents the phase velocity dispersion curves in such a plate (E = 70.8 GPa, ν = 0.34, and ρ = 2700 kg/m${}^{3}$) computed using the DISPERSE software package version 2.0.20a [21]. As can be seen in Figure 1, SH${}_{0}$ is the only nondispersive guided wave mode that propagates in plate-like structures.

In order to significantly reduce the signal processing complexity, the inspection frequencies are typically chosen below the cutoff frequency thickness product of the first high-order mode so as to limit the number of propagating modes. Limiting the frequency also has the benefit of limiting the effect of attenuation, hence enabling longer-range inspections [22] (p. 189–190). Moreover, SH waves have low energy leakage in the surrounding medium as compared to Lamb waves, due to the absence of particle motion normal to the plate or pipe.

**Figure 2:**
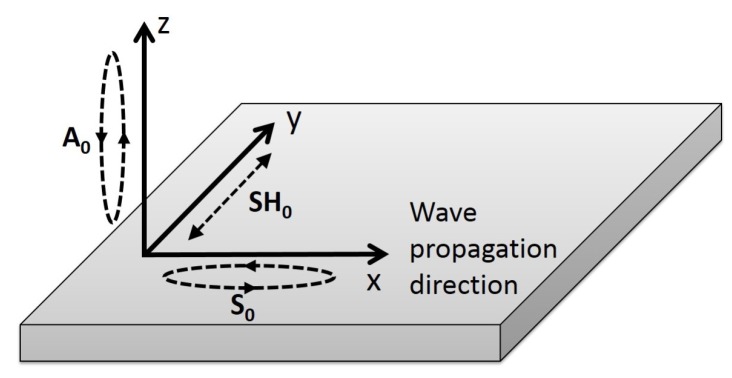
Schematic of the dominant particle motion of each fundamental mode at low frequencies according to the direction of propagation (here in the positive x-direction); ${\mathrm{SH}}_{0}$ has a pure motion polarization regardless of the frequency, while both fundamental Lamb modes have a quasi-pure motion polarization at low frequencies.

In order to generate ultrasonic waves in a medium, a stress must be generated. The generated ultrasonic modes thus depend on the type of stress that is generated in the medium, as well as on the excitability of each mode [23]. The SH${}_{0}$ mode is the only fundamental mode with a particle motion perpendicular to the propagation direction and parallel to the plate; it is also the only one with a single particle motion component. This unique particle motion was shown to be beneficial in monitoring the quality of bonded lap joints [5] or the mechanical properties of brazed lap joints [10]. Figure 2 illustrates the dominant particle motion of each of the fundamental ultrasonic guided wave modes when propagating in the same direction (here the x-axis). Both fundamental Lamb modes exhibit a quasi-pure particle polarization at low frequencies: z-direction for the A${}_{0}$ mode and x-direction for the S${}_{0}$. Consequently, the A${}_{0}$ mode has a high z-direction excitability , and S${}_{0}$ has a high x-direction excitability. On the other hand, SH${}_{0}$ exhibits a pure particle polarization and thus has a high y-direction excitability.

**Figure 3:**
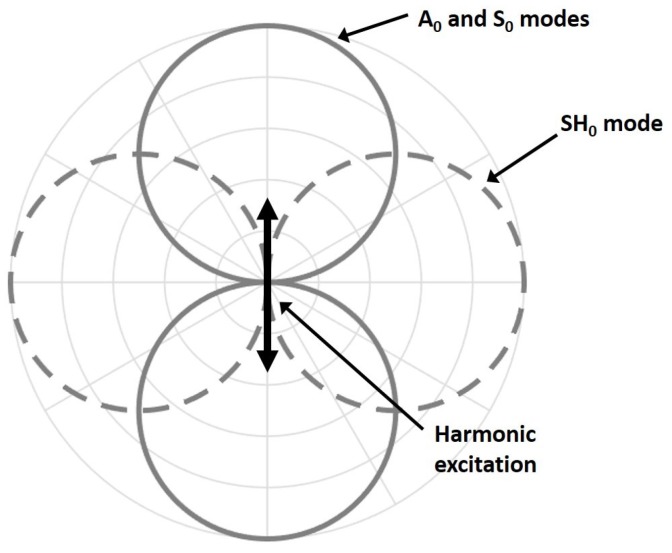
Directivity patterns of a harmonic surface in-plane stress point source. The dashed light-gray lines represent the ${\mathrm{SH}}_{0}$ mode that propagates perpendicular to the excitation direction and the solid light-gray lines represent the $A_{0}$ and $S_{0}$ modes, which propagate along the direction of excitation.

Figure 3 shows the directivity pattern of the acoustic field generated by an in-plane point source for each fundamental mode [24]. Unfortunately, as illustrated in Figure 3, the excitation required for the SH${}_{0}$ mode results in the generation of both fundamental Lamb modes in the excitation direction. Referring to Figure 2, S${}_{0}$ is approximately 6 dB higher than A${}_{0}$ for a low-frequency in-plane excitation. For such a point source excitation, the generated Lamb modes follow a dipole pattern with lobes in opposition of phases, while the SH${}_{0}$ mode also follows a dipole pattern but with lobes in-phase [25].

**Figure 4:**
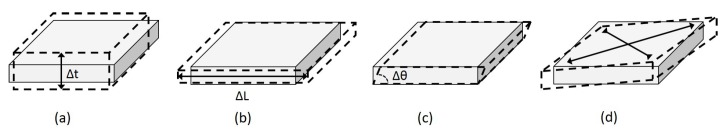
Displacement modes of a piezoelectric plate: (**a**) thickness-extensional displacement mode; (**b**) length-extensional displacement mode; (**c**) thickness-shear displacement mode, and (**d**) face-shear displacement mode.

**Figure 5:**
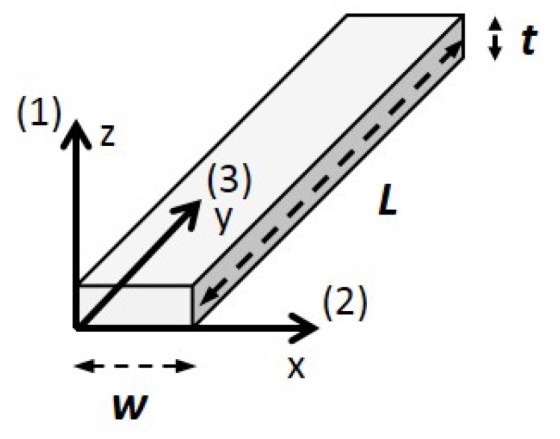
Piezoelectric conventional coordinate system used to describe the piezoelectric samples purchased from Boston Piezo-Optics. The sample is poled along the third axis, and the electrodes are on the faces normal to the first axis. Geometric parameters *L*, *w*, and *t* will be used as reference in this paper.

Piezoelectric transducers use the converse piezoelectric effect to generate strain when subjected to an electric field. Therefore, the first step in the development of a transducer is to choose the right material that will generate the desired strain. Four fundamental vibrational modes can be used to obtain a surface shear strain in a plate-like piezoelectric sample: thickness-extensional mode via Poisson effects, length- or width-extensional mode, thickness-shear mode, and face-shear mode [26] (p. 15). Figure 4 illustrates these four modes of deformation. The two preferred modes are the thickness-shear mode and the face-shear mode because of the absence of extensional displacement due to Poisson effects in the axis of the generated SH waves. Assuming a polycrystalline ferroelectric material with the geometry shown in Figure 5, the thickness-shear mode is preferred to the face-shear mode, as the principal propagation directions of both Lamb modes and SH${}_{0}$ mode will coincide with the crystal axes unlike the face-shear mode [27]. This is of major importance to ensuring that the optimization criteria—which will be discussed later—can be applicable. The thickness-shear mode using the previous statements can be obtained via six different combinations of piezoelectric constants $d_{iij}$ and poling directions, which correspond to every non-equal *i* and *j* subscripts. Using this notation, subscript *i* denotes the corresponding sample thickness axis (where the electric field is applied), and subscript *j* denotes the axis corresponding to the length of the sample, with the commonly used axis for piezoelectric polarization direction as the third one. Following this assumption, the ideal material would only exhibit one of the six possible $d_{iij}$ coefficients, with all 17 remaining coefficients equal to zero to excite the desired mode without exciting unwanted ones. Only four crystal classes exhibit this exact configuration: the orthorhombic mm${}^{2}$ class, the tetragonal 4 mm class, and the hexagonal 6 mm and $\bar{6}$ m${}^{2}$ classes [28] (p. 296–301). Very few materials exhibit such stable structures at room temperature. Fortunately, lead zirconate titanate (PZT) belongs to the tetragonal 4 mm class in these conditions and is thus a great candidate. PZT-5H was chosen for this work because it has the greatest $d_{15}$ coefficient among all existing PZT compositions. The PZT-5H piezoelectric constant matrix is:

(1) $$d=\left|\begin{array}{cccccc} 0 & 0 & 0 & 0 & 730 & 0\\ 0 & 0 & 0 & 730 & 0 & 0\\ -265 & -265 & 530 & 0 & 0 & 0 \end{array}\right|10^{-12}\frac{C}{N},$$

and its detailed properties can be found from the supplier [29].

Geometric parameters of the piezoelectric sample can be optimized in order to maximize the generation of the plane SH mode while minimizing the generation of the fundamental Lamb modes. Two geometric parameters are of great interest for the generation of ultrasonic guided waves: the first is the ratio of the dimension of the piezoelectric element in the direction of propagation of a mode to its wavelength—this dimension being the width *w* for the SH${}_{0}$ mode and the length *L* for the Lamb modes (Figure 5). This parameter governs the level of resulting energy under the transduction area of the transducer as a function of frequency for a given mode. Thus, the resonance frequencies—characterized by a maximum of amplitude—will be obtained for wavelengths that satisfy nλ/2 = *d* (with *n* = 1, 3, 5, etc.), where *d* is specific to each mode and corresponds to the transducer dimension in the mode propagation direction. Similarly, the antiresonance frequencies—characterized by a minimum in amplitude—will be obtained for wavelengths that satisfy nλ = *d* (with *n* = 1, 2, 3, etc.) [30] (p. 216).

The second important geometric parameter is the ratio of the propagating wavelength to the sample’s dimension perpendicular to the propagating axis, *L* for the SH${}_{0}$ mode and *w* for both fundamental Lamb modes. This ratio controls the aperture and the number of generated lobes. According to the far-field propagation theory, if the ratio λd is greater than or equal to one, the source will act as a dipole (Figure 3), and only the main lobe will exist [30] (p. 87). On the other hand, when this ratio becomes smaller than one, the aperture tends to reduce, and side lobes of lower amplitude appear [31]. Thus, to obtain a plane SH${}_{0}$ wave, the length of the transducer *L* should be greater than—or at least equal to—the desired propagating SH${}_{0}$ wavelength. Ideally, in the case of Lamb waves, both their wavelengths should be equal to or less than the width of the transducer *w*, thereby increasing their main lobe directivity; consequently, only their side lobes of lower amplitude would interfere with the SH${}_{0}$ wave field. Table 1 summarizes both optimization criteria and describes the optimal situation for each mode in order to obtain the highest SH${}_{0}$ purity level with a single piezoelectric element.

## 3. Simulations

Because poling is a critical and difficult step, large PZT-5H rectangular plates (*L* = 25.4 mm, *w* = 50 mm, *t* = 1 mm) poled by the manufacturer in the *L* direction were bought from Boston Piezo, Inc. The 25.4 mm length was chosen, as it was the maximum available poled dimension. The minimum frequency of the transducer was thus limited to 213 kHz, as the S${}_{0}$ mode wavelength is greater than 25.4 mm below this frequency ($v_{{\mathrm{ph}}_{S0}}=5429$ m/s at 213 kHz, thus $\lambda_{S_{0}}$ = 25.4 mm). Below that frequency, it is impossible to have an integer multiple of the wavelength with a piezoelectric element length of 25.4 mm. Classical bulk ultrasonic transducers are often designed as thickness resonators to operate in the megahertz range where the wavelengths over the transduction surface dimensions ratio are necessarily smaller than unity, thus fully satisfying the plane wave approximation. However, it is not the case in this study, since frequencies under the first cutoff frequency are used, which results in comparatively long wavelengths. Therefore, the transducer length was established at its highest possible value (25.4 mm), in order to maximize the SH${}_{0}$ plane wave generation.

Since the x-direction excitability of the S${}_{0}$ mode is greater than that of A${}_{0}$ in the frequency range of interest (both exhibiting a quasi-pure polarization as previously discussed), effort was put towards the minimization of the S${}_{0}$ mode using the first criterion [23]. With a fixed length of 25.4 mm, four frequencies between 213 kHz and the first high-order mode cutoff frequency fully satisfy this criterion for the S${}_{0}$ mode: 213 kHz, 425 kHz, 631 kHz, and 829 kHz. These frequencies correspond, respectively, to one-to-four S${}_{0}$ wavelengths in the 25.4 mm length of the transducer. The corresponding length-to-A${}_{0}$ wavelength ratios are then 3.33, 5.17, 6.86, and 8.44. At these four frequencies, the corresponding widths to maximize the SH${}_{0}$ transmission are 7.4 mm, 3.7 mm, 2.5 mm, and 1.9 mm.

### 3.1. Analytical Simulations

Wave propagation finite element simulations are known to be computationally intensive, as a minimum of ten elements per wavelength are required (the maximum element size is 0.3 mm for 829 kHz simulations, as the smallest wavelength, A${}_{0}$, is 3 mm). 3D finite element modeling is necessary in order to be able to simulate both Lamb and SH mode propagation [25]. For this reason, the two geometrical criteria were first explored using an analytical cylindrical wave propagation model, and then a subset of the analytical simulations was validated against a finite element model. This analytical model is based on the Huygens’ superposition principle combined with the dipole pattern assumption of an in-plane point source. Using this model, the displacement field due to a point source excitation can be obtained using:

(1) $$u\left(r,\theta ,t\right)=\frac{cos(\theta )}{2\pi }\sum_{m}\int_{-\infty }^{+\infty }A\left(\omega \right)E_{m}\left(\omega \right)H_{0}^{\left(1\right)}\left(k\left(\omega \right)r\right)e^{i\omega t}d\omega ,$$

where *u* is the three-dimensional resulting displacement field as a function of the angle θ, the distance *r*, and time *t*; A(ω) is the complex amplitude of the input signal, $E_{m}\left(\omega \right)$ is the in-plane excitability of the *m*th mode, $H_{0}^{\left(1\right)}$ is the zeroth-order Hankel function of the first kind, and the *cos*(θ) term represents the dipole directivity of the SH mode (this term changes to sin(θ) for Lamb modes). The excitation area was discretized using a minimum of four point sources per propagating wavelength to ensure convergence of the solution. Finally, the total displacement field was obtained by summing the contribution of each point source based on the Huygens’ principle [25,32].

**Figure 6:**
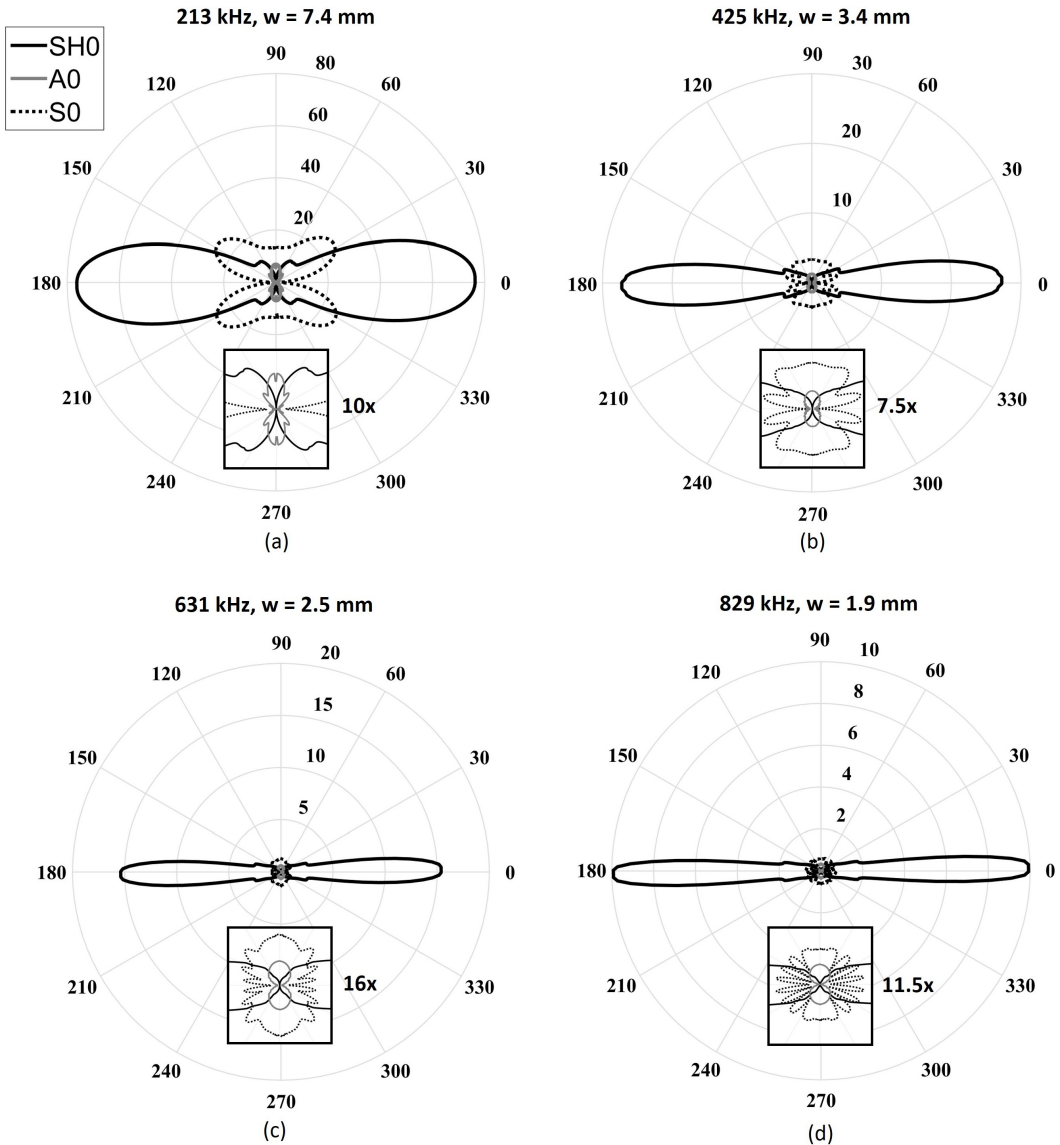
Maximum amplitude wavenumber directivity patterns of the fundamental modes obtained from the analytical model for a fixed length (*L* = 25.4 mm) and fixed thickness (*t* = 1 mm) with a varying five-cycle Hann-windowed toneburst excitation centered at (**a**) 213 kHz (*w* = 7.4 mm); (**b**) 425 kHz (*w* = 3.7 mm); (**c**) 631 kHz (*w* = 2.5 mm); and (**d**) 829 kHz (*w* = 1.9 mm). The solid black lines represent the ${\mathrm{SH}}_{0}$ mode, the dashed black lines represent the $S_{0}$ mode, and the solid gray lines represent the $A_{0}$ mode.

Figure 6 illustrates the directivity patterns of the four previously determined geometries obtained using the analytical model. The directivity patterns consist of the normalized amplitudes of each mode as a function of the angle. The amplitudes were obtained by performing a spatial fast Fourier transform (FFT) to separate the modes based on their wavenumbers. The excitability of each mode, $E_{m}\left(\omega \right)$, to a shear excitation on the surface of a plate was determined using both the DISPERSE software package and finite element simulations for every desired frequency. A five-cycle Hann-windowed toneburst centered at each frequency was used as the excitation signal. As expected, the SH${}_{0}$ wave field became narrower with increasing frequency for a fixed length. Additionally, as expected, in all cases, the A${}_{0}$ beam was at a minimum of 26 dB under SH${}_{0}$ due to its poor in-plane excitability below the cutoff frequency thickness product of the first high-order mode. The S${}_{0}$ directivity changed considerably over the studied frequency range. Indeed, at the lowest frequency (213 kHz), only the main lobe could be observed, while side lobes appeared gradually from 425 kHz to 829 kHz, with a focused main lobe. As observed, the desired minimization effect due to the ratio between the length of the piezoelectric element and the wavelength of the Lamb modes was only effective at angles close to the direction of propagation. Increasing the frequency led to a reduction of the beam opening angle of the main lobe (minimized using the first criterion), but it also led to the appearance of lower-amplitude side lobes, which were unfortunately not being minimized using the same criterion.

### 3.2. Finite Element Simulations

**Figure 7:**
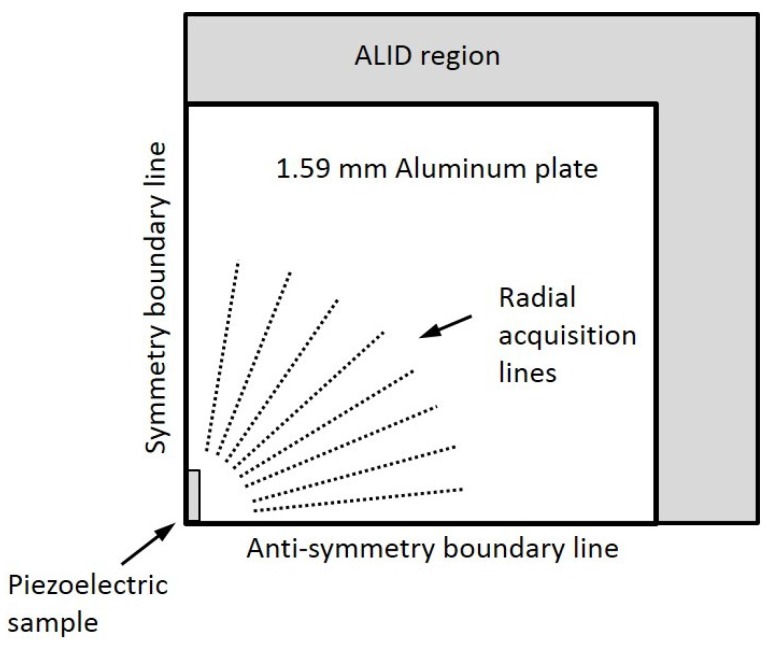
Reduced finite element scheme of a quarter plate. The symmetry boundary line is used in the Lamb modes propagation direction, while the anti-symmetry boundary line is used in the SH mode propagation direction. Absorbing layers with increasing damping (ALID) region are used to avoid reflections from edges. Data is acquired at several angles over radial lines.

**Figure 8:**
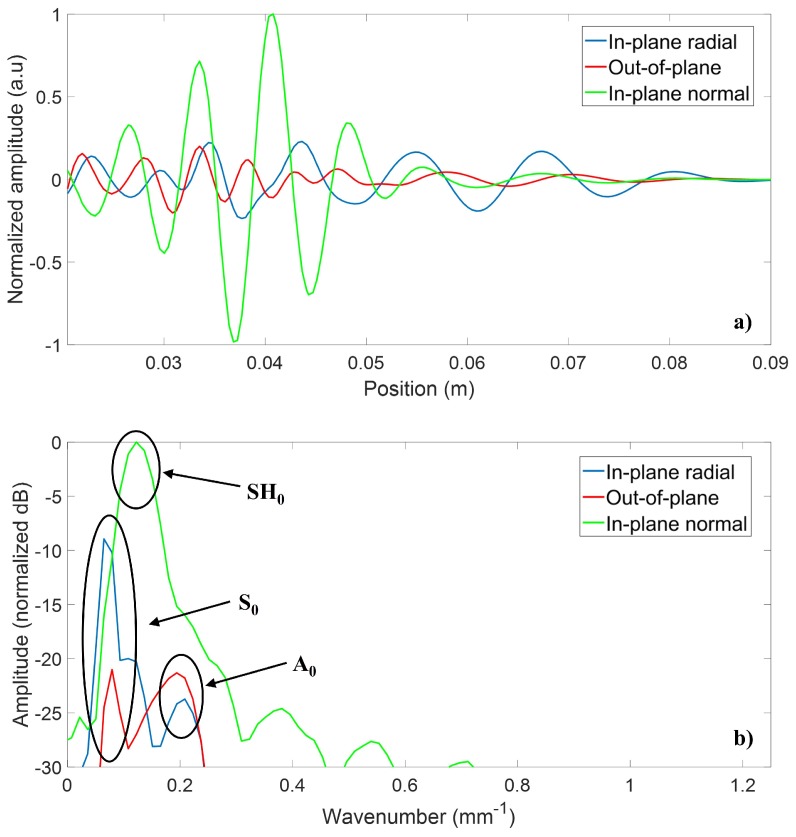
Typical (**a**) normalized spatial trace and (**b**) spatial fast Fourier transform (FFT) used to obtain the different directivity patterns in both simulation types. The blue line represents the in-plane radial displacement component (mainly associated with the $S_{0}$ mode), the red line represents the out-of-plane displacement component (mainly associated with the $A_{0}$ mode), and the green line represents the in-plane normal displacement component (exclusively associated with the ${\mathrm{SH}}_{0}$ mode).

Wave propagation simulations were then performed using the Abaqus finite element software package to validate the results obtained with the analytical model. A 3D finite element model was used, combining the absorbing layers with increasing damping (ALID) region as well as symmetry and anti-symmetry boundary lines to avoid undesired wave reflections and to reduce the model size, and thus the computation time [33]. The ALID region length was chosen to be at least five times the longest propagating wavelength in order to fully absorb the propagating waves, and thus avoid unwanted reflections from the edges [33]. A schematic of the model is presented in Figure 7. A minimum of ten elements per the smallest propagating wavelength ($\lambda_{A_{0}}$ = 3 mm @ 829 kHz) were used, and the time step was chosen to be smaller than the greatest wave velocity divided by the element size to avoid numerical errors [34]. Spatial fast Fourier transforms were performed over several radial lines at constant angles, after which the directivity patterns were then plotted based on the amplitude at the wavenumber corresponding to the three modes of interest. Each mode was separated according to its dominant displacement component: in-plane radial for the S${}_{0}$ mode, in-plane normal for the SH${}_{0}$ mode, and out-of-plane for the A${}_{0}$ mode. Figure 8 illustrates both a typical spatial trace and a typical spatial FFT that were used to obtain the different directivity patterns.

**Figure 9:**
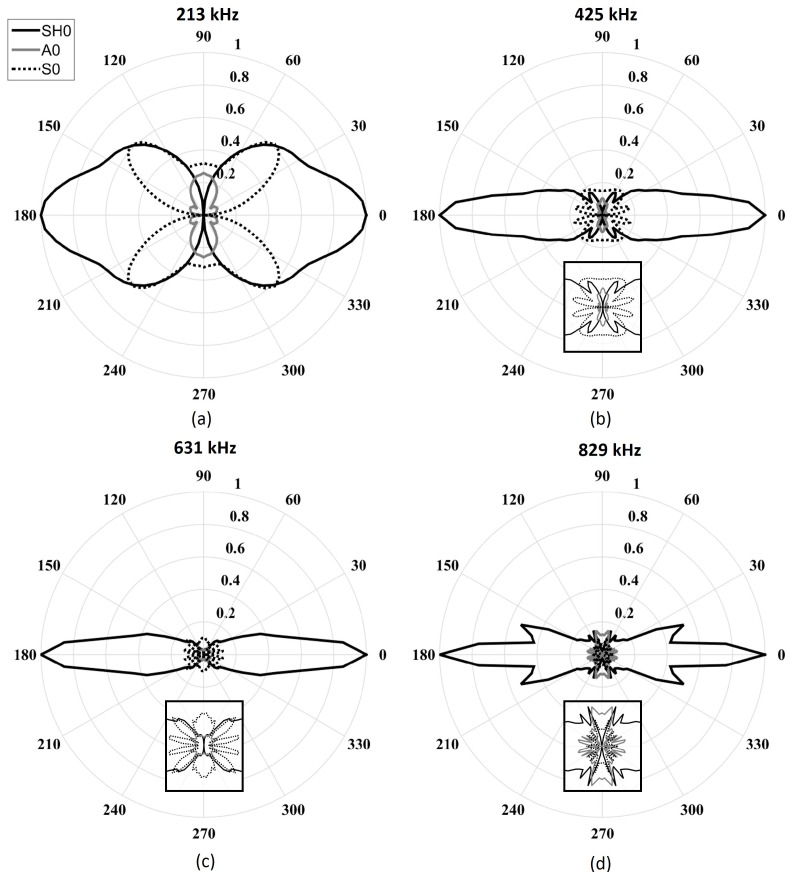
Maximum amplitude wavenumber directivity patterns of the fundamental modes obtained by finite element simulations for a fixed length (*L* = 25.4 mm) and fixed thickness (*t* = 1 mm) with a varying five-cycle Hann-windowed toneburst excitation centered at (**a**) 213 kHz (*w* = 7.4 mm); (**b**) 425 kHz (*w* = 3.7 mm); (**c**) 631 kHz (*w* = 2.5 mm); and (**d**) 829 kHz (*w* = 1.9 mm). The solid black line represents the ${\mathrm{SH}}_{0}$ mode, the dashed black line represents the $S_{0}$ mode, and the solid light-gray line represents the $A_{0}$ mode.

Figure 9 illustrates the directivity patterns obtained by finite element simulations. The lowest frequency design reveals that the SH${}_{0}$ main directivity lobe opening angle was underestimated using the analytical model, while S${}_{0}$ and A${}_{0}$ directivity lobes appeared to be of the same shape, but at twice the amplitudes in this case. It should be noted that in all configurations, the amplitude of the A${}_{0}$ directivity lobe was greater than that obtained using the analytical model. This observation can be explained by the effect of the moving mass of the transducer, which creates a pendulum effect; this phenomenon was neglected in the analytical model, as the physical aspect of the transducer was not taken into account. This mass displacement stressed the plate in the out-of-plane direction. Indeed, this pendulum-like movement added an undesired normal stress that explains the higher amplitude of the A${}_{0}$ mode for which the out-of-plane excitability was high at low frequencies (Figure 2). The last two configurations studied—631 kHz and 829 kHz—present similar directivity lobes as the analytical ones, except the SH${}_{0}$ side lobes amplitudes were greater at 829 kHz .

Finally, a frequency response analysis of the different configurations was performed using Comsol Multiphysics 5.0 software. The first electromechanical resonance frequency—in this case the length-shear fundamental—appeared at around 450 kHz and was almost independent of the width and thickness of the sample, and slightly depended on the mass of the piezoelectric element. For this reason, the design frequency was chosen to be 425 kHz, as the desired shear motion corresponded perfectly to this resonance mode of the piezoelectric sample.

## 4. Experimental Validation

**Figure 10:**
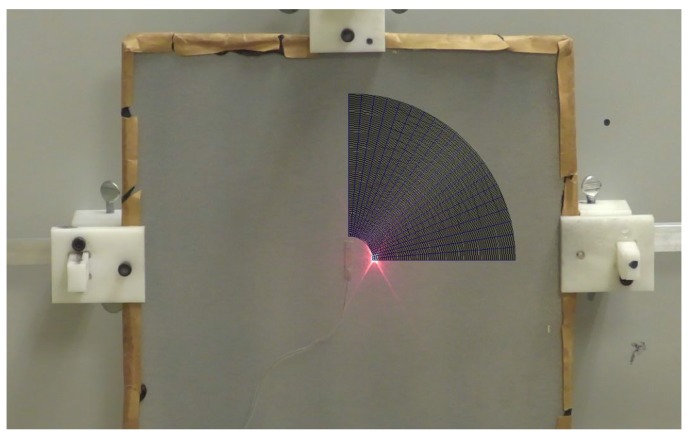
Experimental setup used to validate the 425 kHz configuration; a Polytec PSV-500-3D-M laser Doppler scanning vibrometer system was used to extract the displacement field on a circular array of points. Contacts were ensured using silver-loaded epoxy (Chemtronics Conductive Epoxy).

**Figure 11:**
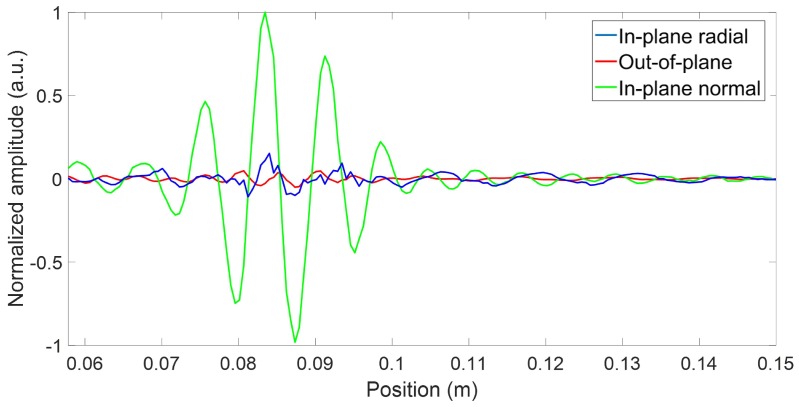
Typical normalized experimental spatial trace used to obtain the directivity pattern based on modes wavenumber maximum amplitude using spatial FFT. The blue line represents the in-plane radial displacement component (mainly associated with the $S_{0}$ mode), the red line represents the out-of-plane displacement component (mainly associated with the $A_{0}$ mode), and the green line represents the in-plane normal displacement component (exclusively associated with the ${\mathrm{SH}}_{0}$ mode).

**Figure 12:**
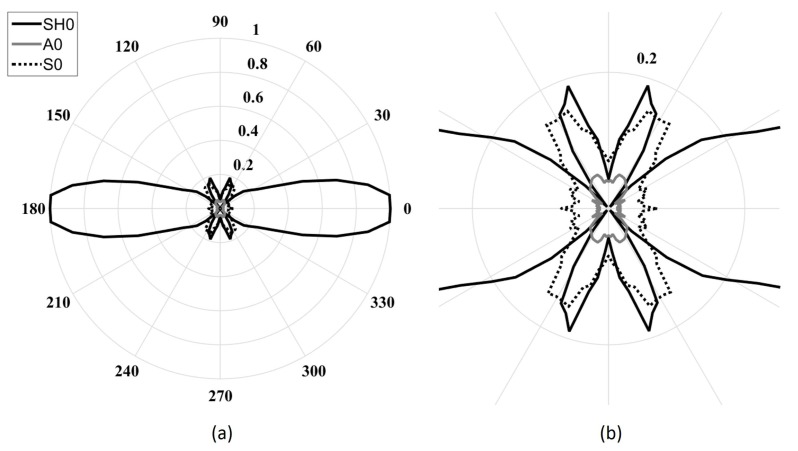
(**a**) Maximum amplitude wavenumber experimental directivity patterns of the fundamental modes obtained using a Polytec PSV-500-3D-M laser Doppler scanning vibrometer system for a 3.7 mm-wide transducer with a synthesized five-cycle toneburst excitation centered at 425 kHz; (**b**) 4× zoom on the fundamental Lamb modes. The solid black lines represent the ${\mathrm{SH}}_{0}$ mode, the dashed black lines represent the $S_{0}$ mode, and the solid light-gray lines represent the $A_{0}$ mode.

The experimental validation was conducted for the 425 kHz configuration, which is a 25.4 mm-long, 3.7 mm-wide, and 1 mm-thick PZT-5H rectangular plate geometry. The effect of the thickness was assumed to be negligible, as the thickness-shear and the thickness-extensional resonance frequencies appeared significantly far from the centered 425 kHz design frequency. The only effect of the piezoelectric sample thickness seen in this frequency range for such a low thickness to other geometric parameters was the ultrasonic wave attenuation due to the material damping. However, it can be neglected for such a thin layer of PZT. Figure 10 illustrates the experimental setup that was used for validation. Pseudo-random excitation signals under 1 MHz were used combined with a NOVO UAP-8400 voltage amplifier. To match the simulation’s excitation conditions, the five-cycle Hann-windowed toneburst was synthesized from the equivalent bandwidth transfer functions. Samples were supplied with chrome/gold-deposited electrodes, and the top contact and the bonding to the plate were ensured using silver-loaded epoxy (Chemtronics Conductive Epoxy). Sufficient plate reflectivity in order to perform the measurements was ensured using metallic particle-loaded paint. The 3D displacement components along 21 equally-spaced radial lines over a quarter of a circle were measured using a Polytec PSV-500-3D-M laser Doppler scanning vibrometer system (LDV). The number of points and their spacing on each acquisition line was set to have a minimum of five points per wavelength for every propagating mode at the desired frequency. These displacements were obtained in the form of transfer functions between the input voltage and the LDV measured displacement field. Figure 11 illustrates a typical spatial trace obtained when converting the pseudo-random excitation to the five-cycle Hann-windowed toneburst. The same procedure as in the simulations was then used to obtain the transducer directivity pattern for all three modes based on their wavenumber; that is, 2D FFT (one dimension—time—being already performed by the LDV) to isolate the propagating modes based both on their frequencies and wavenumbers [35]. Figure 12 illustrates the experimental wavenumber amplitude directivity patterns hence obtained. The experimental results agreed very well with both simulation types. In fact, S${}_{0}$ had an even lower amplitude in its propagation direction than in both simulation cases, and the A${}_{0}$ wave field had the expected shape and was—as expected from the simulations—of small amplitude (refer to Table 2). The desired SH${}_{0}$ wave field appeared to have the same aperture as predicted using both simulations, but with more constant amplitudes at angles very close to the propagation axis.

**Figure 13:**
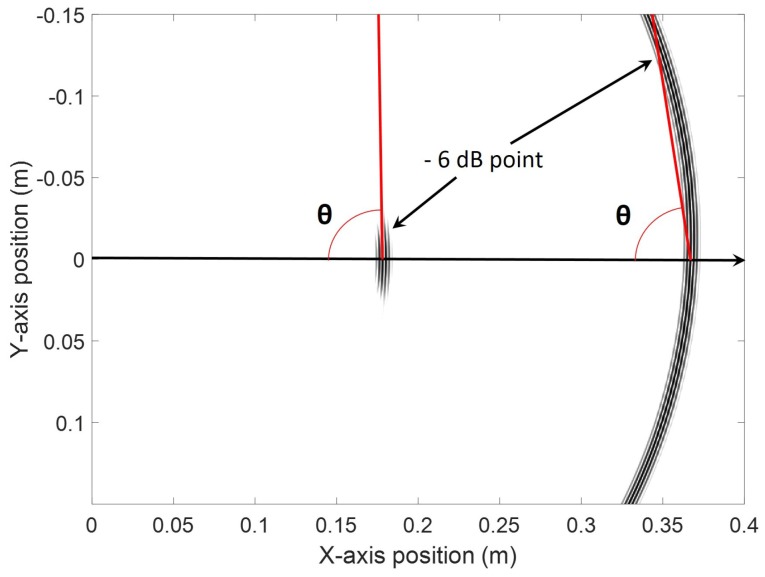
Schematic of how the 6 dB wavefront angle with respect to the propagation direction was calculated. As an illustration, the SH${}_{0}$ mode was used at two different times of propagation and for two different transducer geometries at a constant frequency.

Table 2 presents the ratios in decibels of the maximum amplitudes of the Lamb modes to the maximum amplitude of the SH_0_ mode at any angle for all designed configurations in both simulation cases, as well as for the experimental validation of the 425 kHz design. In addition, Table 2 presents the planarity of the 6 dB SH_0_ main wavefront based on the angle that it makes with respect to its propagation direction as illustrated in Figure 13. Trends between both analytical and finite element simulations appear to be consistent. The planarity of the wavefront increased with frequency because the corresponding second criterion’s ratios were reducing. The experimental 6 dB wavefront orientation for the design at 425 kHz was measured to be 86.73°.

## 5. Conclusions

This paper presented a procedure of geometric optimization for a low-frequency piezoceramic transducer optimized to generate a plane SH_0_ wave. While the proposed procedure was for applications where transducers are directly bonded to the structure, it could easily be extended to different applications. A study of piezoelectricity and the different vibrational modes led to the choice of PZT-5H as the optimal material for the transducer design. The geometry of the piezoelectric sample was first theoretically optimized, and then validated using both simulations and experimentally. It was shown that by optimizing the geometric parameters of a rectangular transducer, directive SH_0_ mode could be generated at a high level of purity compared to both fundamental Lamb modes. The generated amplitude was shown to be at least 16.4 dB above both fundamental Lamb modes in any direction. Moreover, the level of purity of the SH_0_ mode was shown to be 23.0 dB within an aperture of 20° of its axis of propagation for a designed frequency centered at 425 kHz. Finally, the experimental 6 dB wavefront angle was found to be 86.73° with respect to its propagation direction, confirming the planarity of the generated wavefront. This optimization process is of significant importance for future structural health monitoring applications in the oil and gas or aerospace industries, where plane features such as welds or riveted joints are safety-critical.

## Figures and Tables

**Table 1:** Optimal ratios of both criteria for each fundamental mode (*n* = 1, 3, 5, etc., and *m* = 1, 2, 3, etc.).

Wave Mode	SH_0_	S_0_	A_0_
**First criterion ratio**	$w=\frac{n\lambda }{2}$	L=mλ	L=mλ
**Second criterion ratio**	$\frac{\lambda }{L}\leq 1$	$\frac{\lambda }{w}\leq 1$	$\frac{\lambda }{w}\leq 1$

**Table 2:** Ratios of the maximum amplitude of Lamb modes to the maximum amplitude of the SH_0_ mode and orientation (*θ*) of the SH_0_ 6 dB wavefront with respect to its propagation direction for all designed geometries in both simulation cases and for the experimentally validated 425 kHz design.

	A_0_/SH_0_ (dB)	S_0_/SH_0_ (dB)	SH_0_ Wavefront *θ*
**Analytical 213 kHz**	−20.7	−9.2	82.52°
**Analytical 425 kHz**	−26.2	−18.0	86.51°
**Analytical 631 kHz**	−27.9	−21.5	87.44°
**Analytical 829 kHz**	−29.5	−22.8	88.55°
**FE 213 kHz**	−11.9	−4.2	82.84°
**FE 425 kHz**	−19.6	−14.6	86.61°
**FE 631 kHz**	−24.5	−17.2	87.73°
**FE 829 kHz**	−16.4	−19.9	88.60°
**Experimental 425 kHz**	−25.7	−16.4	86.73°
